# The Prognostic Value of Pentraxin-3 in COVID-19 Patients: A Systematic Review and Meta-Analysis of Mortality Incidence

**DOI:** 10.3390/ijms24043537

**Published:** 2023-02-10

**Authors:** Anna Paola Capra, Alessio Ardizzone, Giuseppe Pantò, Irene Paterniti, Michela Campolo, Lelio Crupi, Raffaele Squeri, Emanuela Esposito

**Affiliations:** 1 Department of Chemical, Biological, Pharmaceutical and Environmental Sciences, University of Messina, Viale Ferdinando Stagno d’Alcontres, 98166 Messina, Italy; 2 Department of Biomedical and Dental Sciences and Morphofunctional Imaging, University of Messina, Via Consolare Valeria 1, 98125 Messina, Italy

**Keywords:** Pentraxin-3 (PTX3), COVID-19, SARS-CoV-2, mortality, immunopathology

## Abstract

Over the last three years, humanity has been facing one of the most serious health emergencies due to the global spread of Coronavirus disease (COVID-19). In this scenario, the research of reliable biomarkers of mortality from COVID-19 represents a primary objective. Pentraxin 3 (PTX3), a highly conserved protein of innate immunity, seems to be associated with a worse outcome of the disease. Based on the above, this systematic review and meta-analysis evaluated the prognostic potential of PTX3 in COVID-19 disease. We included 12 clinical studies evaluating PTX3 in COVID-19 patients. From our research, we found increased PTX3 levels compared to healthy subjects, and notably, PTX3 was even more augmented in severe COVID-19 rather than non-severe cases. Moreover, we performed a meta-analysis to establish if there were differences between ICU and non-ICU COVID-19 patients in PTX3-related death. We combined 5 studies for a total of 543 ICU vs. 515 non-ICU patients. We found high significative PTX3-related death in ICU COVID-19 hospitalized individuals (184 out of 543) compared to non-ICU (37 out of 515), with an overall effect OR: 11.30 [2.00, 63.73]; *p* = 0.006. In conclusion, we probed PTX3 as a reliable marker of poor outcomes after COVID-19 infection as well as a predictor of hospitalized patients’ stratification.

## 1. Introduction

SARS-CoV-2 virus belongs to the Coronaviridae family, a subfamily of Orthocoronavirinae and structurally can be defined as an enveloped positive-sense single-stranded RNA virus [1,2,3]. During the last three years, the spread of SARS-CoV-2 led to one of the most severe public health emergencies in the world, known as the Coronavirus disease (COVID-19) pandemic. 

COVID-19 infectious disease, first reported in the city of Wuhan (China) in December 2019, has rapidly spread to the rest of the world, and the World Health Organization (WHO) declares the COVID-19 pandemic on 11 March 2020 [4]. The most frequent clinical manifestations, after an incubation period of 2 to 14 days (median 5 days), are heterogenous and may significantly vary in gravity between patients, ranging from completely asymptomatic disease to cases associated with mild or moderate flu-like symptoms such as cough, fever, myalgia, headache, dyspnea, sore throat, diarrhea, nausea, vomiting, loss of taste and smell. Lethality has been estimated to average 5%, but the risk of severe/critical infection and death increases with age and in presence of comorbidities, such as cardiovascular disease, diabetes, hypertension, chronic respiratory disease, cancer, etc. [5].

Moreover, many genetic and non-genetic conditions in each individual may influence the outcome, thus providing people at high risk of developing a certain severity during the infection. 

To date, some of the main clinical problems related to COVID-19 have been partially mitigated by the refinement of the pharmacological agents available. In particular, the prompt development of vaccines and the rapid prophylaxis of a large chunk of the population has been in this regard an almost unprecedented achievement in modern medicine. The literature data enquiring about the efficacy of vaccines (51 studies from 14 countries), highlighted how vaccination against COVID-19 provided a significant, powerful, and prolonged protective effect both for hospitalization, hospitalization in the intensive care units (ICUs), and death from COVID-19 [6]. Despite all these achievements, the COVID-19 pandemic still poses several significant challenges. The most relevant concern related to the continuous persistence of the pandemic is represented by the outbreak of new viral variants with greater infectivity or mortality, or less responsivity to the currently available therapeutic agents [7]. Efforts are still required to better understand the pathophysiological mechanisms of COVID-19 as well as the immune response against an ever-changing virus, to identify new pharmacological tools, and find reliable prognostic predictors of the disease and/or of the occurrence of long-term alterations.

Pentraxin-3 (PTX3), an essential component of humoral innate immunity, has been recognized in resistance to selected pathogens and regulation of inflammation [8,9]. PTX3 belongs to the pentraxin superfamily, an evolutionarily conserved group of proteins with essential roles in the recognition of self and non-self-antigens. The similarity with short pentraxin C-reactive protein (CRP) prompted investigations into the utility of PTX3 as a marker in various human conditions of infectious or inflammatory origin. 

In contrast to CRP, produced essentially by the liver in response to interleukin (IL)-6 during the acute phase response [10], PTX3 is rapidly produced by several cell types, including myeloid cells, endothelial cells, and respiratory epithelial cells, particularly in response to IL-1, tumor necrosis factor, microbial molecules, and tissue damage [8,9]. Local production by different cell types at inflammatory sites and release of the preformed protein by neutrophils in response to primary pro-inflammatory cytokines or microbial recognition explain the rapidity of PTX3 increase in these conditions. 

Among its roles in the organism, PTX3 is involved in the recognition and elimination of pathogens, acting as an opsonin, and therefore stimulating phagocytosis [11]. PTX3 significantly amplifies neutrophil response [12], thus contributing to the enhancement and prolongation of the inflammatory response. This activity strictly depends on the activation of the alternative complement pathway, mainly through the binding of the complement receptor 3 (CD11b/CD18) [12]. PTX3 can interact with the complement component 1q (C1q) [13], following a calcium-independent mechanism, thereby inducing the activation of the classical complement pathway. Thus, while PTX3 may contribute to the clearance of pathogens, it can also exacerbate the severity of pathological inflammatory responses [14]. The organism’s aberrant response to infection can lead to a state such as systemic inflammatory response syndrome (SIRS), which, in severe cases, can lead to sepsis. To confirm this, high plasma concentrations of PTX3 have been associated with disease severity and mortality in various pathological conditions [15,16]. PTX3 has been shown to act as a biomarker of disease activity in inflammatory conditions involving the vascular bed, from atherosclerosis to vasculitis [16,17] and immunity alterations too. 

The immune system is crucial in protecting the organism and effectively responding to viral respiratory infections and, in this regard, SARS-CoV-2 infection makes no exception. Immune cells are provided with a set of specific extracellular and cytosolic receptors, i.e., Toll-like receptors (TLRs), RIG-like receptors (RLRs), NOD-like receptors (NLRs), and absent in melanoma 2 (AIM2)-like receptors (ALRs), which allows them to early respond to aspecific inflammatory signals, such as pathogen-associated molecular patterns (PAMPs) and damage-associated molecular patterns (DAMPs). Following the interaction with these viral stimuli, a specific immune response occurs, with the release of a plethora of soluble factors, cytokines, chemokines, and the complement system [18]. Several pieces of evidence have been provided showing that immunological memory of SARS-CoV-2 may last even beyond 6 months post-infection [19,20]. Indeed, studies specifically evaluating immune memory to SARS-CoV-2 revealed that memory CD4^+^ T cells and memory CD8^+^ T cells could be detected 6 months after infection in 90% and 70% of convalescent patients, respectively, and memory B cells in nearly all patients in the same time frame [19,20]. In addition to the above immunological memory, there is evidence that SARS-CoV-2 infection may induce long-lasting immune response abnormalities remaining detectable 11 months after infection [21], also causing a clear shift in CD4+ and CD8+ cells at 3 months post-infection [22]. This clinical picture, known as post-COVID-19 syndrome, includes a variety of neurological, cardiovascular, autoinflammatory, renal, and endocrine manifestations, which can persist for several months [23]. 

Considering the PTX3 pattern, it seems to be increased during COVID-19 disease, and such high levels were linked to a greater risk of mortality [24]. Thus, PTX3 could be a possible predictor of patients’ clinical course following the infection. Other studies propose PTX3 as a discriminating factor of hospital stratification, indicating how its high levels would be related to a more probable ICU admission than to other wards. 

However, the specific impact of PTX3 on COVID-19 progression is still unclear due to the lack of large multicenter trials as well as the few studies in the literature because of the short time from the pandemic. Hence, whether PTX3 has prognostic value and evident clinical significance still needs to be defined. 

This systematic review aimed to assess PTX3 value as a diagnostic and prognostic factor in COVID-19 infection and also discuss, through quantitative method (meta-analysis) the related increase in mortality in different hospitalized patients. The accurate evaluations of PTX3 levels in COVID-19 patients can be useful in providing a new perspective for clinical practice and follow-up.

## 2. Methods

### 2.1. Methods

The PubMed (MEDLINE) and Embase (OVID) bibliographic databases were scrutinized for the literature search. We used The Preferred Reporting Items for Systematic Review and Meta-analysis Protocols (PRISMA-P) as guidelines for reporting the research strategy of the studies. APC and AA performed the bibliographic search based on the eligibility criteria summarized in Table 1 and considering only articles written in English. 

Two content experts (MC and EE) designed the search strategy and supervised the study. No geographic exclusion criteria or temporary restrictions were imposed. 

In PubMed (MEDLINE) and Embase (OVID), terms related to PTX3, and COVID-19 were examined by using specific keywords indicated in Table 2. 

### 2.2. Study Selection

Following PubMed (MEDLINE) and Embase (OVID) search, we removed duplicates then two review authors (APC and AA) individually screened the titles and abstracts of all records identified to remove articles that were not pertinent. Consequently, we examined the full-text articles to select the records that corresponded most closely to the eligibility criteria; conflicting opinions were resolved through the mediation of a third review author (EE). The data extraction from the included articles was performed by two authors (APC and AA). From the 12 included studies we collected the following data: title, author(s), year of publication, study catchment area (i.e., geographic zone), study population (i.e., different populations of COVID patients or healthy subjects, ICU vs. non-ICU).

### 2.3. Assessment of Risk of Bias

The quality of eligible records described in this systematic review was individually evaluated by two reviewers (APC and AA) using the Newcastle Ottawa scale (NOS; see Supplementary Table S1) as previously described [25,26]. Based on this judgment, the study’s value was classified as low, medium, or high. Dissimilarities in score assignments were resolved through the involvement of a third review author (EE). After the authors’ evaluations, none of the articles were considered at high risk of bias.

### 2.4. Data Synthesis Methods for Meta-Analysis

For statistical analysis in the quantitative synthesis, we used an odds ratio (OR) measure and the random-effects model with the Mantel–Haenszel method. At the end, we obtained pooled estimates of the variant effect (OR) with the associated 95% confidence interval (CI). The heterogeneity was estimated through a graphical examination of the forest plots and then assessed using the I^2^ statistic as previously described [25,26]. Review Manager (Rev Man. Version 5.4. Copenhagen: The Nordic Cochrane Centre, The Cochrane Collaboration, 2014) was used to perform the meta-analysis of the obtained data.

## 3. Results and Discussion

### 3.1. Findings from Systematic Search

**Figure 1:**
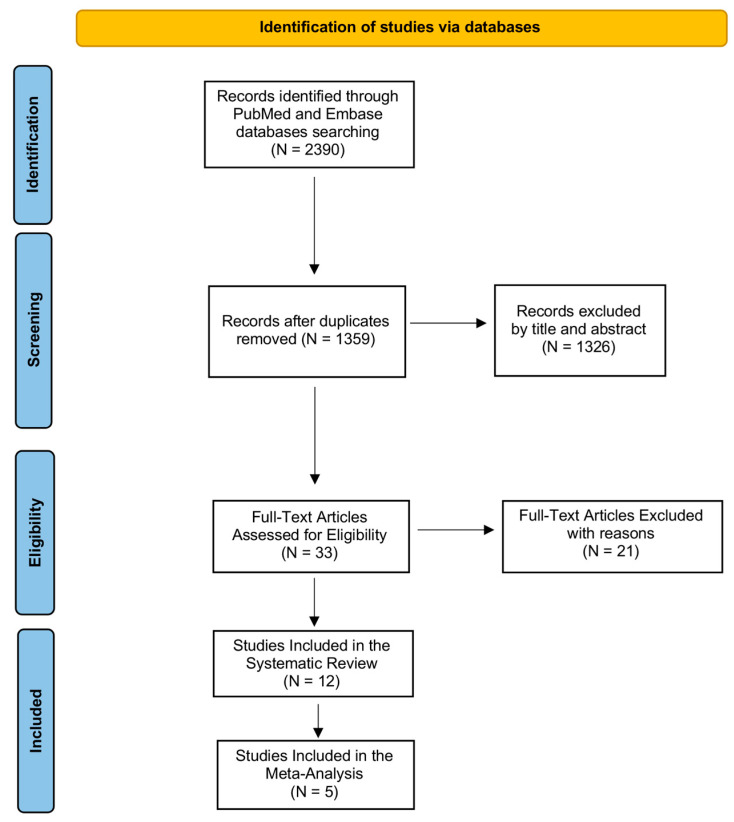
PRISMA flow diagram. The figure describes every step of search strategy and screening, all process was performed according to PRISMA-P guidelines.

We illustrated the entire screening process in Figure 1 through the PRISMA-P flowchart. During the first step of the search by using the above-mentioned keywords, we identified 2390 records in PubMed (MEDLINE) and Embase (OVID) databases. After removing duplicates, we obtained 1359 records, which were analyzed by title and abstract in order to evaluate their eligibility. We excluded 1326 articles after reading the title and abstract because they were not relevant to our review question. Then, we examined the full text of 33 articles for eligibility, excluding 21 records as they did not match the established inclusion and exclusion criteria. We included 12 studies in our systematic review as shown by the PRISMA Flowchart reported in Figure 1 since evaluated PTX3 in COVID-19 patients, and specifically comparing COVID-19 patients to healthy subjects or analyzing PTX3 levels in the stratification of COVID-19 hospitalized. 

We also performed a meta-analysis on the incidence of mortality related to PTX3 levels in different hospitalized COVID-19 patients. For this purpose, from 12 studies included in the systematic review, we selected only 5 records that assessed PTX3 levels in ICU compared to non-ICU patients. 

### 3.2. Evaluation of Included Studies in the Systematic Review

In each included study PTX3 levels were assessed by laboratory testing at hospital admission. Overall, PTX3 values were significantly higher in COVID-19 patients than in healthy subjects, thus denoting an alteration of the immunoinflammatory process following infection in which PTX3 could act as the main driver. Furthermore, analyzing the stratification of hospitalized patients (in the studies in which it was presented), we noted that PTX3 was considerably increased in ICU patients requiring mechanical ventilation and adequate care considering the life-threatening state. PTX3 levels were slightly lower in patients admitted to other wards. PTX3 values are summarized in Table 3 while the detailed description of the included studies is presented in the Discussion section.

### 3.3. Meta-Analysis of Mortality Incidence among Different COVID-19 Populations

From the screening performed to identify studies eligible for meta-analysis, only five records matched the inclusion criteria (quantitative assessment of PTX3 in ICU vs. non-ICU hospitalized patients). 

In the studies included, PTX3 had been proposed as a possible prognostic factor in COVID-19 patients, and its levels had been ascertained through routine laboratory tests. 

Those five articles elucidated PTX3 as a predictor of the degree of hospitalization, thereby its high values were often related to patients requiring mechanical ventilation and care at the ICU.

Considering these assumptions, we wanted to investigate through quantitative analysis the degree of mortality among COVID-19 subjects admitted to the ICU, comparing them with non-ICUs.

**Figure 2:**
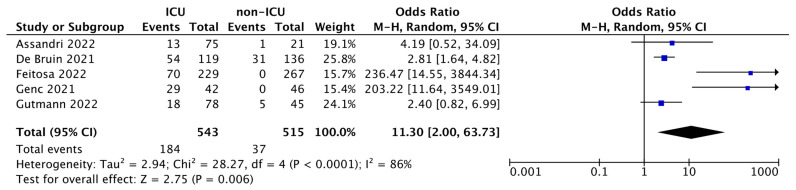
Forest plot of the studies included in the quantitative synthesis. The forest plot describes the mortality incidence between ICU and non-ICU patients in which PTX3 levels were assessed. The death of patients is referred as events compared to the total number of subjects. Squares display the effect estimate (ORs) with the size of each blue square corresponding to the weight given to each study in the meta-analysis. Horizontal lines represent the 95% CIs corresponding to each effect estimate. The black diamond represents the overall effect of intervention with its width representing the overall 95% CI. The I^2^ statistic represents a measure of heterogeneity. Overall effect OR: 11.30 [2.00, 63.73]; *p* = 0.006.

As shown in the forest plot (Figure 2), ORs range between 2.40–236.47, and although the heterogeneity was considerable (I^2^ = 86%), the total OR was 11.30 (95% CI: 2.00–63.73) and the test for overall effect was *p* = 0.006. This result revealed a powerful statistical significance, indicating the markedly increased mortality rate of ICU patients compared with non-ICUs, thus demonstrating that high levels of PTX3 in COVID-19 patients admitted to the ICU in addition to foresee the degree of hospitalization are also a reliable predictor of patients’ death. 

PTX3 emerged as a strong independent death predictor better than conventional biomarkers such as CRP and IL-6. In fact, performing an overall assessment of the five studies included in the meta-analysis, only PTX3 can be proposed as an accurate prognostic factor, leading to consistent and statistically significant results, unlike CRP and IL-6 which were variable and sometimes weakly associated with mortality. These data are certainly worthy of further clinical investigations.

### 3.4. Discussion

SARS-CoV-2 is the agent responsible for the COVID-19 pandemic disease. The virus can target the respiratory system, spreading from human to human via respiratory droplets from infected subjects [39]. From a few localized cases at the end of 2019, the disease has rapidly spread throughout the entire planet in the first months of 2020. As of 13 January 2023, the official data from the WHO reports 661.545.258 confirmed cases of COVID-19, including 6.700.519 deaths. 

One of the primary mechanisms of severe clinical complications is the aberrant inflammatory response resulting from the rapid viral replication in the alveolar cells, which triggers an initial Th1 response and a subsequent massive lung tissue infiltration of macrophages and neutrophils as well as the secretion of pro-inflammatory cytokines [40]. This pathological process, known as “cytokine storm”, contributes to severe and life-threatening pulmonary and extrapulmonary complications, ultimately leading to a state of multiorgan failure [41,42]. A proposed pathogenic hypothesis suggests that a prolonged inflammatory state associated with cytokine hypersecretion may be responsible for a state of moderate latent inflammation, which, in turn, could lead to chronic inflammation-related symptoms [43]. The assessment of humoral innate immunity molecules, such as PTX3, in the context of COVID-19 disease, could be useful in monitoring the prognosis.

Therefore, this systematic review aimed to analyze and summarize the prognostic value of PTX3 in COVID-19 patients, also elucidating the related clinical outcomes in different COVID-19 hospitalized subjects.

Although the spread of COVID-19 is limited to just three years, we found several articles that analyzed the role of the PTX3 protein in affected patients. The eligible advances indicated the potential significance of PTX3 as a reliable prognostic factor in COVID-19 patients as well as in different degrees of disease severity, such as ICU compared to non-ICU subjects. Brunetta et al. assessed the presence of PTX3 in patients with COVID-19 [28]. The authors carried out the study on two cohorts of patients, the first consisting of 96 subjects admitted to Humanitas Clinical and Research Center (Milan, Italy), the second independent cohort of 54 individuals admitted to the ASST Papa Giovanni XXIII (Bergamo, Italy). Increasing plasma concentrations of PTX3 were discovered in 96 COVID-19 patients (median 17.3 ng/mL; *p* < 0.0001) together with a considerable increase in IL-6 contents (*p* = 0.017), while CRP evaluation did not show prominent results (*p* = 0.082). Indeed, among the various inflammatory markers analyzed, PTX3 emerged as a strong independent predictor of 28-day mortality in multivariate analysis [28]. PTX3 levels appeared higher in dead patients compared to surviving individuals (median 39.8 ng/mL), but also in ICU patients compared to ward patients [28]. These data were further validated in a cohort of 54 patients, PTX3 resulted in a better predictor of mortality (*p* = 0.026) than CRP (*p* = 0.203) and IL-6 (*p* = 0.099) [28]. Genc et al. evaluated the predictive value of PTX3 in COVID-19 pneumonia [31]. The study was performed on 88 confirmed COVID-19 patients of which 59 were later found to be survivors and 29 non-survivors [31]. Very high levels of PTX3 were found in all COVID-19 individuals with a median of 3.66 ng/mL; in addition, such levels were significantly greater in non-survivors compared to survivors (*p* = 0.045) [31]. Kukla and colleagues estimated several biochemical parameters, including PTX3, using immunoenzymatic methods [34]. For this purpose, 70 confirmed COVID-19 patients (43 females and 27 males) and 20 healthy volunteers (10 females and 10 males) were enrolled in the study [34]. In the first analyses between COVID-19 (2337.7 pg/mL) and non-COVID-19 (2030.9 pg/mL) subjects, slight PTX3 differences were identified, although not statistically significant (*p* = 0.55) [34]. While, according to previous results, consistent PTX3 serum concentrations were detected in the 9 COVID-19 patients needing ICU care (4768.9 pg/mL) compared to 61 patients that did not require it (2278.2 pg/mL) [34]. However, the authors reported no difference in PTX3 concentration between pneumonia patients and healthy subjects.

In the ICU cohort analyzed by Gutmann et al., PTX3, determined by ELISA, emerged as a protein positively associated with COVID-19 mortality [32]. The population was composed of 123 COVID patients, of which 78 were ICU and 45 were non-ICU. Among the 78 in the ICU, 60 survived and 18 died after infection [32]. The control group consisted of 55 non-COVID-19 patients, among these, 25 were hospitalized in the ICU unit while 30 were non-ICU [32]. Considering the analysis of the ICU hospital ward, in non-surviving COVID-19 patients, PTX3 was considerably elevated (4.93 ng/mL) compared to levels of surviving patients that were 2.16 ng/mL [32]. PTX3 drives COVID-19 infections toward more chronic and more disabling states. Accordingly, its increase leads to both a greater risk of developing COVID-19 pneumonia (pneumonia: 2.92 ng/mL vs. no pneumonia: 2.28 ng/mL) and a greater risk of hospitalization in the ICU wards (ICU care: 4.77 ng/mL vs. no ICU care: 2.30 ng/mL) [35]. Moulana and colleagues found augmented levels of PTX3 in the serum of life-threatening COVID-19 patients. Of the total 98 subjects enrolled in the study, 14 patients were hospitalized in the ICU, 59 patients were hospitalized in the non-ICU wards and 25 subjects represent the healthy control group [37]. From data obtained by ELISA kit performed on serum samples, ICU patients had higher levels of PTX3 when compared to non-ICU patients (1957 ± 1769 pg/mL vs. 1220 ± 1784 pg/mL) or healthy subjects (1957 ± 1769 pg/mL vs. 275 ± 167 pg/mL) [37]. An overlapping trend was detected in the study of Assandri et al., indeed, patients admitted to ICU presented higher PTX3 concentrations compared to non-ICU patients (median value 35.86 ng/mL vs. 10.61 ng/mL) [27]. Control individuals showed a median of 2.30 ng/mL [27]. Furthermore, the authors highlighted the higher accuracy of PTX3 compared to CRP, lactate dehydrogenase (LD), and ferritin in identifying ICU patients. In addition, from laboratory test results of enrolled patients, PTX3 emerged as one of the most reliable, unlike other routinely used inflammatory markers such as IL-6 (*p* = 0.551).

Thus, the unfavorable outcome in the general ward and the ICU was associated with changes in concentrations of PTX3, as deepened in a prospective cohort study by de Bruin et al. [29]. 

PTX3 was a reliable biomarker both in predicting unfavorable outcomes in the general ward and also associated with death in the ICU [29]. On the other hand, no significant variation in CRP levels was found between survivors and non-survivors in the ICU cohort (*p* = 0.24), highlighting the high variability of this biomarker in predicting mortality in COVID-19 issues.

Hence, this study contributes to the characterization of the clinical course of patients with PTX3-related severe COVID-19. In this complex clinical picture, several pathways come into play including chemotaxis and interleukin production, but also endothelial dysfunction, the complement system, and immunothrombosis. All these factors, if not kept under control through adequate prophylaxis, make COVID-19 a highly unpredictable disease, which is based on interindividual susceptibility ranging from asymptomatic to respiratory failure or death. Measurement of PTX3 within 4 days of admission proved to be a predictive value of mechanical ventilation and 30-day mortality compared with clinical parameters and other markers of inflammation [33]. As Hansen and colleagues stated, in death patients the median PTX3 concentration upon admission was 19.5 ng/mL (IQR: 12.5–33.3) versus 6.6 ng/mL (IQR 2.9–12.3) (*p* < 0.0001) for survivors [33], as well IL-6 levels resulted increased in non-survivors compared to survivors (*p* < 0.0001). Else, no significant alteration in CRP values was detected (*p* = 0.18).

The most recent advances, in addition to confirming the previous insights, have highlighted the involvement of PTX3 in the activation and regulation of the complement system while reiterating its important role in the pathogenesis of COVID-19. PTX3 plasma levels were significantly associated with COVID-19 severity and mortality (*p* < 0.05) [30]. The severe group had higher PTX3 levels (median: 987.0 pg/mL) when compared to the moderate group (median: 570.5 pg/mL) (*p* = 0.0004) [30]. PTX3 levels at admission were observed to be 3.3 times higher in patients who died than in those who survived (2233 pg/mL [*n* = 25] versus 663.2 pg/mL [*n* = 144], *p* < 0.0001) [30]. Moreover, Feitosa et al. suggested as PTX3 levels were significantly correlated with IL-6, IL-8, IL-10, CRP, total leukocytes, neutrophil-to-lymphocyte ratio, urea, creatinine, ferritin, length of hospital stay, and higher respiratory rate (*p* < 0.05) [30]. PTX3 was associated with the risk of patients’ death (per 10 ng/mL, HR 1.08; 95%CI 1.04–1.11; *p* < 0.001) and in the death/mechanical ventilation ratio (HR 1.04; 95%CI 1.01–1.07; *p* = 0.011), independently of other predictors of in-hospital mortality, including age, Charlson Comorbidity Index, D-dimer, and CRP as assessed by Lapadula and colleagues [36]. Patients with PTX3 levels above the optimal cut-off of 39.32 ng/mL had significantly higher mortality than the others (55% vs. 8%, *p* < 0.001) [36]. Indeed, in multivariate analysis of death, PTX3 was the most significant factor compared to CRP or D-dimer [36].

Furthermore, higher PTX3 plasma levels were found in 14 patients with subsequent thrombotic complications [36]. Sulicka-Grodzicka et al., in a manner consistent with previous work, found higher levels of PTX3 in severe COVID-19 patients than in non-severe COVID-19 [38]. The authors evaluated the sequence of inflammatory responses in acute COVID-19 through a 28-day follow-up, discovering that the resolution of inflammation in the group of moderate/severe SARS-CoV2 infection was associated with decreasing PTX3 serum concentrations [38]. The authors performed a time course analysis of PTX3 on day 1, day 7, and day 28 after infection [38]. Their results revealed a constant and progressive decrease in PTX3 from day 1 to day 28 [38]. On other hand, analyzing inflammatory markers between non-severe COVID-19 and severe COVID-19, PTX3 was significant only on day 1, unlike TNFα and IL-1β, which were increased also 28 days post-infection [38]. 

In a similar manner, Hansen et al. reported PTX3 levels over time for COVID-19 patient survivors and non-survivors for 14 days following hospital admission, with slightly different kinetics [33]. Considering that most of the included studies do not carry out a 28-day follow-up analysis, we believe that future studies are needed to establish a long-term course of PTX3 in order to provide more consistent conclusions.

PTX-3 levels usually increased after 6–8 h of the inflammatory process and may result in the release of certain cytokines that may cause a prolonged cytokine storm [31]. Otherwise, it is necessary to wait more than one and a half days for an increase in other biomarkers such as CRP to trigger inflammation [31]. From these statements, appears clear the early upregulation of PTX3 among other inflammatory markers, thus supposing how its decrease could be associated with COVID-19 resolution. If we comprehensively examine our findings, PTX3 appears to be the most consistent biomarker in determining the disease outcome when compared to other known biomarkers, such as CRP or IL-6. Indeed, unlike PTX3, the assessment of CRP or IL-6 among the various cohorts of COVID-19 patients has not always led to statistically significant results, thus revealing the high heterogeneity of results concerning these two biomarkers.

The combination of 5 studies in meta-analysis resulted in a total of 543 ICU vs. 515 non-ICU patients. The meta-analysis carried out shows that there is a significant increase in mortality in ICU patients (184 out of 543) compared to non-ICU patients (37 out of 515); OR: 11.30 (95% CI: 2.00–63.73; *p* = 0.006). This aspect, as stated by the included studies, is directly correlated to the increase in serum PTX3, which therefore, in addition to being useful in predicting the patient’s hospital stratification, also constituting a predictor of death. 

However, some limitations need to be addressed in the present study, firstly the few included studies. The collected findings can be subjected to potential bias due to different variables. We included studies based on COVID-19 cohorts’ patients that can differ regarding age, sex, ethnicity, viral variants, and other clinical complications, as well as individual pharmacological treatments. For instance, among the included records, the time of sample collection was heterogenous and the experimental procedures with different sensitivity may have been used to detect PTX3 levels. 

Rather, none of the included studies presented gender-specific stratification, so we could not perform subgroup analysis by using this parameter. Moreover, some potential biomarkers were not analyzed, so the direct comparison with other conventional or not conventional biomarkers was not included in the present study.

Despite several factors that can impact the clinical outcomes of COVID-19 disease, our question is still valid in defining if PTX3 can be a useful prognostic factor to monitor disease severity and mortality.

In fact, although it had previously been demonstrated that circulating PTX3 levels presented a non-significant difference between COVID-19 patients in the ICU and non-ICU [44], their correlation with PTX3-related death appears quite different, as supported by our results. Considering the comparable level of comorbidity between the two groups in most of the included studies, we retain that future clinical evaluations should investigate possible PTX3-driven immunoinflammatory cross-talks which could be different between ICU and non-ICU patients. Meanwhile, our findings contribute to the clinical overview of the role of PTX3 in COVID-19 unfavorable outcomes, also promoting the potential benefits of the immunotherapy approach for COVID-19 patients similar to what was previously exposed in the oncology field [45].

## 4. Conclusions

Taken together, the data discussed in this systematic review and examined in the meta-analysis highlight PTX3 as a reliable biomarker in predicting COVID-19-related death. In the 12 eligible records, the high expression of this protein was a predictor of poor clinical outcomes, often being correlated with ICU admissions. Analyzing PTX3-related death through the quantitative method we detected a strong statistical significance in ICU compared to the general ward patients (*p* = 0.006). Given the importance of PTX3 in driving the early immunoinflammatory reaction leading to a more severe form of COVID-19 disease, its analysis by laboratory tests should be highly recommended by clinicians. Future research could deeply characterize this biomarker in different COVID-19 clinical pictures by evaluating gender differences and pathophysiological variables together with immunoinflammatory signaling cross-talks. In conclusion, the collected evidence supported the development of PTX3-targeting drug therapies as a promising approach to moderate the inflammatory response in COVID-19 patients. 

## Figures and Tables

**Table 1:** Description of eligibility criteria.

Inclusion Criteria	Exclusion Criteria
Observational studies, case–control studies, case reports, cross-sectional studies, and cohort studies Papers indicating quantitative PTX3 levels in COVID-19 patients compared to healthy subjects or evaluating PTX3 in ICUs hospitalized compared to non-ICUs or analyzing PTX3 in non-survivors compared to survivors	Editorials, letters, reviews, guidelines, abstracts and paper conferences, systematic reviews and meta-analyses, and ongoing studies Articles not written in English

**Table 2:** Keyword combinations used during the search strategy.

Pentraxin-3	COVID-19
Pentraxin, Pentraxins, Pentaxin, Pentaxins, Pentraxin-3, Pentraxin3, Pentraxin 3, PTX, PTXs, PTX-3, PTX3.	COVID-19, COVID19, COVID-19 Virus, COVID-19 Viruses, COVID-2019, SARS-CoV-2, SARS-CoV-2 Infection, Coronavirus, Coronaviruses.

**Table 3:** Table summarizing the articles included in the systematic review and related PTX3 levels in different subjects.

First Author and Year of Publication	Main Outcome	Time Point of Samples Collection	Methods	References
Assandri et al., 2022	Healthy controls: median 2.30 ng/mL COVID-19 patients: median 31.32 ng/mL (*n* = 96) ICU patients: median 35.86 ng/mL (*n* = 75) non- ICU patients: median 10.61 ng/mL (*n* = 21)	Samples were obtained within the first 24 h following hospital admission.	PTX3 serum concentrations were measured by a commercial ELISA kit according to manufacturer’s indications.	[27]
Brunetta et al., 2021	Whole COVID-19 sample: median 17.3 ng/mL (IQR 10.1–39.8) (*n* = 96) ICU patients median 21.0 ng/mL (IQR 15.6–46.3) (*n* = 52) non-ICU patients median 12.4 ng/mL (IQR 6.12–20.2) (*n* = 44) Patients who died from COVID-19: median 39.8 ng/mL (IQR 20.2–75.7 ng/mL) (*n* = 22; 14/22 from ICU; 8/22 from medical ward) Surviving patients: median 15.3 ng/mL (IQR 8.2–21-3)	Venous blood samples were collected during the first 5 d after hospital admission (mean ± s.d., 2.1 ± 1.6 d).	PTX3 plasma concentrations were measured by a sandwich ELISA developed in-house, by personnel who were blinded to patient characteristics. Measurements were taken from distinct samples, and each sample was tested in duplicate.	[28]
de Bruin et al., 2021	Changes in concentrations between the first blood sampling and the subsequent retesting of PTX3 were associated with unfavorable outcome and death (see article Hazard Ratio in ICU vs. non-ICU patients)	For the current analysis, patients were selected when a blood sample was available within 2 days of ward and/or ICU admission.	PTX3 levels were measured using magnetic Luminex assay. A total of 642 measurements (ward: 156; ICU: 486) of sufficient quality were used for the statistical analysis.	[29]
Feitosa et al., 2022	Patients with severe COVID-19: median 987.0 pg/mL (*n* = 86) Patients with moderate COVID-19: median 570.5. pg/mL (*n* = 83) Patients who died from COVID-19: median 2233.0 pg/mL (*n* = 25) Surviving patients: median 663.2 pg/mL (*n* = 144)	Samples were collected within 24 h of hospital admission.	PTX3 was measured by ELISA in the plasma of patients included in the study following manufacturer’s instructions.	[30]
Genç et al., 2020	Whole COVID-19 sample: median 3.66 ng/mL (IQR 0.9–27.9) (*n* = 88) Patients who died from COVID-19: median 3.91 ng/mL (IQR 1.9–23.2) (*n* = 29) Surviving patients: median 3.3 ng/mL (IQR 0.9–27.9) (*n* = 59)	Patients whose serum could be separated for PTX-3 at the time of hospitalization were included in the study.	Human PTX3 levels were analyzed with a commercial human ELISA kit following manufacturer’s instructions.	[31]
Gutmann et al., 2021	Non-survivors: median 4.93 ng/mL (IQR 2.78–6.20) Survivors: median 2.16 ng/mL (IQR 1.41–3.64)	Serial blood sampling was performed within 24 h of admission to ICU and thereafter three measurements were taken during week 1, week 2, and again before discharge.	PTX3 were assessed by ELISA kit according to the manufacturer’s instructions.	[32]
Hansen et al., 2022	Whole COVID-19 sample: median 8.6 ng/mL (IQR 4.1–18.3) (*n* = 126) Patients who died from COVID-19: median 19.5 ng/mL (IQR 12.5–35.3) (*n* = 34) Surviving patients: median 6,6 ng/mL (IQR 2.9–12.3) (*n* = 92) This study also included a validation cohort of 112 pts treated with remdesivir and dexamethasone. Whole COVID-19 sample: median 29.9 ng/mL (IQR 13.7–56.1) Survivors: median 26.5 ng/mL (IQR 12.2–51.0) (*n* = 96); Non survivors: median 56.5 ng/mL (IQR 30.9–96.4 (*n* = 16))	A blood sample was drawn within 4 days of admission and for some individuals during follow-up. If more than one sample was drawn from the same patient, the first sample was used in the primary analysis.	Plasma PTX3 levels were quantified by ELISA. Specifically, PTX3 content serum samples were assessed by correlating sample duplicates to a standard curve.	[33]
Kukla et al., 2021	Whole COVID-19 sample: median 2337 pg/mL (IQR 1935.1–3211.5) (*n* = 70) Healthy volunteers: median 2030.9 pg/mL (IQR 1767.4–3499.5) (*n* = 20) ICU patients: median 4768.9 pg/mL (IQR 2896.8–8394.5) (*n* = 9) non-ICU patients: median 2278.2 pg/mL (IQR 1876.8–3106.2) (*n* = 61)	Serum samples were obtained from the peripheral blood collected at the hospital admission before any treatment was applied.	PTX3 serum concentrations were assessed in duplicate using an immunoenzymatic method with commercially ELISA kits.	[34]
Kuśnierz-Cabala et al., 2021	Whole COVID-19 sample: median 2.37 ng/mL (IQR 2.02–3.48) (*n* = 70) Patients with pneumonia: median 2.92 ng/mL (IQR 2.19–4.77) (*n* = 23) Patients without pneumonia: median 2.28 ng/mL (IQR 1.84–3.21) (*n* = 47)	The samples were collected between day 0 and day 5 after admission to hospital.	Serum concentrations PTX-3 were measured by ELISA using commercially available kits.	[35]
Lapadula et al., 2022	Whole COVID-19 sample: median 21.7 ng/mL (IQR 13.5–58.2) (*n* = 152) Patients who died or required MV: median 68.3 ng/mL (IQR 39.8–115) (*n* = 47) Patients discharged alive free from MV: median 18.0 ng/mL (IQR 11.1–29.1) (*n* = 105) Patients with thromboembolic events: median 51.4 ng/mL (IQR 24.6–94.4) (*n* = 14)	Plasma samples were collected at hospital presentation or within 48 h of admission.	Levels were measured by ELISA kit. Each sample was tested in duplicate.	[36]
Moulana et al., 2021	ICU patients: M ± SD 1957 ± 1769 pg/mL (*n* = 14) non-ICU patients: M ± SD 1220 ± 1784 pg/mL (*n* = 59) Healthy control group: M ± SD 275± 167 pg/mL (*n* = 25)	Time collection was not clearly specified.	The concentrations of PTX3 were measured by ELISA kit. All assays were performed in duplicate according to the manufacturer’s instructions.	[37]
Sulicka-Grodzicka et al., 2022	Patients with non-severe COVID-19: median 5213 pg/mL (IQR 2743–8301) (*n* = 125) Patients with severe COVID-19: median 8781 pg/mL (IQR 5091–12744) (*n* = 129)	The following time points were defined for blood sample collection for the study: during hospitalization on day 1 and day 7, and during the outpatient follow-up period: one month after admission (28 ± 2 days).	A custom ordered xMAP technology Luminex assay (Bio-Techne, Minneapolis, MN, USA) and Luminex 200 fluorescent-based detection system (Luminex, Austin, TX, USA) was used according to manufacturer protocol.	[38]

## Data Availability

Data of this study are available to the corresponding author’s address.

## Supplementary Materials

The supporting information can be downloaded at: https://www.mdpi.com/article/10.3390/ijms24043537/s1.
