# Supplementary material for: The Prognostic Value of Pentraxin-3 in COVID-19 Patients: A Systematic Review and Meta-Analysis of Mortality Incidence
Source: Int J Mol Sci. 2023 Feb 10;24(4):3537. doi: 10.3390/ijms24043537 (PMC9958638; doi:10.3390/ijms24043537)
Supplement: Supplementary file 1 [file ijms-24-03537-s001.zip › ijms-2197357-supplementary.pdf]

RISK OF BIAS Table	SELECTION				COMPARABILITY		OUTCOME			
	Representative of the exposed cohort	Selection of external control	Ascertainment of exposure	Outcome of interest does not present at the start of the study	Comparability of Cohorts		Assessment of outcomes	Sufficient follow-up time	Adequacy of follow-up	
					Main Factor	Additional Factor				
	1	2	3	4	5	6	7	8	9	OVERALL
Assandri et al., 2022	x	x	x		x	x	x	x	x	8
Brunetta et al., 2021	x	x	x		x	x	x	x	x	8
de Bruin et al., 2021	x	x	x		x	x	x	x	x	8
Feitosa et al. 2022	x	x	x		x	x	x	x	x	8
Genç et al., 2020	x	x	x		x	x	x	x	x	8
Gutmann et al., 2021	x	x	x		x	x	x	x	x	8
Hansen et al., 2022	x	x	x		x	x	x	x	x	8
Kukla et al., 2021	x	x	x		x	x	x	x	x	8
Kusnierz-Cabala et al., 2021	x	x	x		x	x	x	x	x	8
Lapadula et al., 2022	x	x	x		x	x	x	x	x	8
Moulana et al., 2021	x	x	x		x	x	x	x	x	8
Sulicka-Grodzicka et al., 2022	x	x	x		x	x	x	x	x	8

Additional file 1. Table with Risk of Bias judgement according NOS score
